# Genome-editing technologies for gene correction of hemophilia

**DOI:** 10.1007/s00439-016-1699-x

**Published:** 2016-06-29

**Authors:** Chul-Yong Park, Dongjin R. Lee, Jin Jea Sung, Dong-Wook Kim

**Affiliations:** Department of Physiology and Brain Korea 21 Plus Project for Medical Science, Yonsei University College of Medicine, Seoul, 03722 Korea

## Abstract

Hemophilia is caused by various mutations in blood coagulation factor genes, including *factor VIII* (*FVIII*) and *factor IX* (*FIX*), that encode key proteins in the blood clotting pathway. Although the addition of therapeutic genes or infusion of clotting factors may be used to remedy hemophilia’s symptoms, no permanent cure for the disease exists. Moreover, patients often develop neutralizing antibodies or experience adverse effects that limit the therapy’s benefits. However, targeted gene therapy involving the precise correction of these mutated genes at the genome level using programmable nucleases is a promising strategy. These nucleases can induce double-strand breaks (DSBs) on genomes, and repairs of such induced DSBs by the two cellular repair systems enable a targeted gene correction. Going beyond cultured cell systems, we are now entering the age of direct gene correction in vivo using various delivery tools. Here, we describe the current status of in vivo and ex vivo genome-editing technology related to potential hemophilia gene correction and the prominent issues surrounding its application in patients with monogenic diseases.

## Introduction

Hemophilia, an inherited bleeding disorder, can be caused by deficiency in various blood coagulation factor proteins. As an X-linked recessive disorder, hemophilia A and B caused by deficiency in *factor VIII* (*FVIII*) and *factorIX* (*FIX*), respectively, are most common. The incidence of hemophilia A (1 in 5000 males) is approximately six times higher than that of hemophilia B (Mannucci and Tuddenham 2001). Based on coagulation factor activity in a patient’s plasma, hemophilia cases can be classified into one of three types: mild (5–30 % activity), moderate (1–5 % activity), or severe (<1 % activity) (Graw et al. 2005). Severe cases show frequent spontaneous bleeding episodes in joints, muscles, and soft tissues, and the symptoms may progress to chronic synovitis, debilitating arthropathy, and other physical disabilities (Chuah et al. 2013).

One current hemophilia treatment option involves intravenous infusion of recombinant FVIII or FIX concentrate. Due to the short half-life of these factors, however, patients with severe hemophilia must receive the treatment 2–3 times per week throughout their lifetimes. Moreover, this remedy is costly, and some patients generate a neutralizing antibody against the infused factor. Because the clotting factor infusion is more a preventive approach for avoiding bleeding episodes than a curative one, a more permanent approach is needed. Gene therapy can potentially fulfill this need, because (1) hemophilia is an inherited monogenic disease, (2) its symptoms can be improved with a 1 % increase of in vivo coagulation activity, and (3) the patient’s phenotype can be improved through a stable supply of therapeutic proteins, such as FVIII and FIX, instead of having cells producing the coagulation factors. The previous studies tested several vector delivery systems targeting various cell types to pursue gene therapy options. Out of these delivery vector systems, adeno-associated virus (AAV)-mediated approach attracts attention, which has big potentiality in safety or delivery efficiency aspect. However, this approach still has limitations in that the expression of therapeutic genes is not permanent, as the viral genome containing therapeutic gene is maintained in a form of episome, an unwanted insertional mutation may be induced, or unintended side effects (e.g., hepatitis) may result from immune responses (High 2011; Chuah et al. 2012).

To overcome these limitations, researchers have investigated the genomic correction of mutated genes via genome-editing (Cox et al. 2015). Targeted genome-editing can be achieved using programmable nucleases, including zinc-finger nuclease (ZFN), transcription activator-like effector nuclease (TALEN), and clustered regularly interspaced short palindromic repeat (CRISPR)/CRISPR-associated protein 9 (Cas9) systems (Kim and Kim 2014) that can induce DSBs at specific genomic loci. Depending on the cell cycle stage and the presence or the absence of donor template, nuclease-induced DSBs are typically repaired by one of two major cellular repair systems: error-prone non-homologous end joining (NHEJ) and error-free homology-directed repair (HDR). NHEJ is a highly efficient repair system which directly ligates two ends, where DSBs is created. During the repairing process by this mechanism, small insertions and deletions (indels) can be induced in the DSB site. On the other hand, HDR is a high-fidelity repair system that occurs when exogenous template or sister chromatids to homologous sequence is involved. Although NHEJ-mediated editing is more efficient than HDR-mediated editing, HDR system may induce largely error-free repairs. Thus, it has been widely used to correct the monogenetic diseases including hemophilia. However, it has been reported that gene correction utilizing NHEJ is also possible in the following cases (Anguela et al. 2013; Park et al. 2015): when it is difficult to prepare a homologous donor (e.g., large chromosomal inversion), and in the case of excluding homologous arm hierarchy to deliver a long therapeutic gene using AAV vector which has relatively high restraints on cargo sizes.

Various genomic rearrangements (indels, targeted large deletions, inversions, duplications, translocations, and gene knock-ins/-outs) may occur during the repair of one or more induced DSBs. In fact, the gene targeting strategy has long been used, but with the dazzling advance of programmable nucleases, the efficiency of targeting has been enhanced dramatically.

These technologies are being adopted actively for in vivo or ex vivo studies correcting mutated genes that cause inherited monogenic diseases, such as hemophilia. Especially, the development of a delivery system for in vivo genome-editing which does not require ex vivo manipulation step is actively in progress. According to Li et al. (2011), nuclease-mediated in vivo editing utilizing AAV vector delivery system can be made in a tissue specific manner. Up to now, AAV vector is being considered as a more promising delivery tool for in vivo editing. Thus, a smaller version Cas9 that can overcome packaging size limitation of AAV vector has been developed (Ran et al. 2015), and its safety was verified through the recently reported AAV-mediated clinical approach (Nathwani et al. 2014). Although safety aspects or high immunogenicity aspects should be improved in the future, lentiviral (Sánchez-Rivera et al. 2014) and adenoviral vectors (Cheng et al. 2014) can also be considered as delivery systems with in vivo editing potentiality. In addition, although cationic lipid-mediated delivery strategy that could deliver a nuclease in a protein form (Zuris et al. 2015) for in vivo editing was reported, further studies should identify whether the donor DNA delivery capacity and the target cell (or tissue) specificity are covered. Here, we discuss the current status of nuclease-mediated genome-editing technology related to hemophilia treatment and relevant issues involving the application to humans.

## In vivo gene correction

The ideal method for curing hemophilia may be the correction or replacement of mutated coagulation factor genes at the genomic level. To this end, researchers are actively conducting in vivo correction experiments combining the concept of nuclease and AAV-mediated gene therapy. Because this strategy does not rely on in vitro cell manipulation, required in ex vivo systems, it is potentially more advantageous than ex vivo approaches.

Li et al. (2011) constructed two hepatotropic AAVs encoding a ZFN and corrective *FIX* cDNA flanked by homology arms and observed that HDR-mediated in vivo correction using systemic co-delivery of two AAV vectors was possible in humanized neonatal hemophilic mice. Their study is highly meaningful, because it was the first successful nuclease–mediated in vivo genome-editing attempt and showed that nuclease–mediated DSBs can be induced in vivo at a high rate (34–47 %). Although AAV was injected intraperitoneally to 2-day-old mice and HDR-mediated targeting efficiency in their liver tissue was only 1–3 %, their activated partial thromboplastin time (aPTT) was shortened significantly suggesting that nearly complete phenotypic correction could be achieved. Subsequent partial hepatectomy experiments showed that therapeutic FIX was stably expressed for up to 30 weeks, indicating that genomic modification can overcome the limitations of conventional gene therapies and is applicable in infants with growing liver tissues.

Anguela et al. (2013) demonstrated that the above method was also effective in adult mice. They injected two versions of AAV (AAV-ZFN and AAV-donor) intravenously into 8-week-old humanized mice and confirmed that circulating FIX was consistently restored to 23 % of the normal level for 60 weeks. Interestingly, they achieved radical reduction of off-target frequency using obligate heterodimeric ZFN. They also found that both HDR- and NHEJ-mediated integration could be induced effectively, suggesting that knock-ins can be induced using NHEJ in quiescent liver tissue or non-replicating cells. Recently, the same research group reported successful correction of *FVIII* and *FIX* genes through NHEJ- or HDR-dependent mechanisms, respectively, in endogenous mouse albumin (m*Alb*) loci (Sharma et al. 2015). An aPTT assay of HDR-dependent, *FIX*-corrected mice revealed a remarkable functional rescue with improved clot forming ability close to the normal level. As the *Alb* locus can be utilized as a safe harbor, it is also highly adaptable for correcting other monogenetic diseases. When an NHEJ-dependent strategy is used in AAV-mediated in vivo correction research, however, AAV-derived inverted terminal repeats integrate together and thus necessitate further determination of unexpected negative effects caused by the repeating sequences. Moreover, non-integrated AAV genome can be detected several months after the injection and cause negative effects, such as off-target mutations and cytotoxicity resulting from the persistent expression of ZFN. The utility of the m*Alb* locus for gene targeting was previously highlighted in another study (Barzel et al. 2015) that used an AAV-*FIX* donor without a nuclease and found that HDR-mediated integration was possible upstream of the stop codon locus of the *Alb* gene. Correction took place in 0.5 % of tested hepatocytes, and FIX expression was restored to approximately 7–20 % of the normal level, although additional testing in large animal models is necessary to assess the method’s applicability in humans. Using a non-nuclease, AAV-*FIX* donor may be significant in terms of safety, because the exclusion of a nuclease hypothetically limits occurrence of problematic off-target mutations. If HDR efficiency could be enhanced further, the method may be applicable in humans.

According to another recent report, distinguished from the strategies targeting a safe harbor locus as described above, in vivo gene correction was made directly for disease-causing point mutation in the endogenous *FIX* locus (Guan et al. 2016). They delivered Cas9- and the donor-encoded DNA as a naked DNA vector to liver tissue by hydrodynamic injection and attained single-stranded DNA oligonucleotides (ssODNs)- and plasmid donor-mediated HDR efficiency of 0.56 and 1.55 %, respectively. Interestingly, a genome-editing efficiency of 0.56 % restored hemostasis in established *FIX*^*Y381D*^ mice. Because such naked DNA is non-immunogenic in vivo, this is considered an in vivo genome-editing strategy with a potential, although this strategy seems difficult to be actually applied to human subjects. In this way, bringing conventional gene therapy tools to the genome-editing field makes editing in vivo systems on a chromosome level possible. In the case of in vivo genome-editing, however, it is currently not possible to sort out cells with an unwanted mutation or conduct genotyping among edited cells. Therefore, a prior thorough examination of the desired nuclease system’s safety and accuracy must be performed. Moreover, additional efforts should be made for patients with antibodies against AAV.

## Ex vivo gene correction

Ex vivo gene correction is another strategy to cure hemophilia. Because transplantation of autologous cells with restored genes can avoid immune rejection and allow genotypic and phenotypic examination before transplanting the cells, patient-specific induced pluripotent stem cells (iPSCs), in particular, are an important resource in regenerative therapies. They have unlimited self-renewal ability, can be grown as single cell-derived clones, and have the ability to be differentiated into different types of cells composing the human body. However, patient-specific iPSC-based therapies must avoid possible residual pluripotent stem cells or unwanted other type of cells remaining in culture, and must detect and eliminate random and relatively rare genetic mutations that could be acquired during multiple cell divisions to prevent possible tumor formation. Despite the tumorigenicity hurdle, iPSC-based therapeutic approaches have shown their potential for regenerative medicine in animal models and a clinical trial for age-related macular degeneration is undergoing (Kimbrel and Lanza 2015; Trounson and DeWitt 2016). Therefore, patient-specific iPSCs have potentially significant utility in ex vivo gene correction.

In early studies, the possibility of nuclease-mediated gene correction was assessed using healthy cells ex vivo because of difficulty in obtaining cells from patients. Lee et al. (2012) prepared a ZFN targeting the intron 1 homology region of *FVIII* and attempted to build an intron 1 inversion model through transfecting HEK-293 cells with normal *FVIII* genome structure. Although inversion efficiency was low, and indels related to NHEJ-mediated repair were observed, the outcome suggested the possibility that genomic inversion can be corrected close to its original state by this nuclease. Such modeling research was also adopted in attempts to use TALEN in normal iPSCs (Park et al. 2014). With the use of TALEN, the inversion efficiency increased to over 1 %, albeit still low. However, the hemophilia phenotype, which could not be shown in HEK-293 cells due to the presence of three X chromosomes, was reproduced. Furthermore, the study showed that therapeutic *FVIII* expression can be rescued at the chromosomal level by reverting an intron 1 inverted segment to its normal orientation using the same nuclease. This finding led to research with patient-derived iPSCs, which determined that large inversion types (e.g., intron 1 and 22 inversions) of *FVIII* can be reverted using CRISPR/Cas9 (Park et al. 2015). Analysis of off-target mutations through deep and whole-genome sequencing in corrected clones revealed no significant mutations. Although FVIII deficiency in mice can be rescued to some degree by transplantation of corrected iPSC-derived endothelial cells, further research is necessary to address the low corrective effect of the cells, including their efficient differentiation to liver sinusoidal endothelial cells (LSECs, known as major FVIII producing cells), optimization of the transplantation site, and the enhanced survival of transplanted cells. The inverted genome structure can be also successfully restored to its normal direction through transfection of the Cas9/sgRNA ribonucleoprotein complex. Because the complex has a short half-life, this approach minimizes unwanted off-target effects and can potentially prevent random insertional mutations of vector DNA, making it a promising strategy for improving ex vivo therapy of monogenic diseases.

A recent study of TALEN-mediated knock-in introduced another strategy for correcting intron 22 inversion in endogenous loci (Wu et al. 2016). Using intron 22 inverted patient-iPSCs, they successfully induced HDR-mediated targeted integration by inserting normal exon 23–26 sequences in frame after an inverted exon 22 locus. Showing a high targeting efficiency (52.9–62.5 %) through antibiotic-based selection, they demonstrated the potential use of this strategy for correcting inversion, deletion, and point mutation genotypes. For example, correcting a point mutation via knock-in with easily preparable ssODNs donors can be used instead of plasmid donors. Low targeting efficiency is expected, because antibiotic-based selection cannot be used, but PCR-based single colony analysis could be performed to obtain scarless clones. Their study confirmed that functional FVIII proteins are secreted by endothelial and mesenchymal stem cells differentiated from corrected iPSCs. However, additional rescue experiments with animal models should be pursued to determine its therapeutic potential.

Knock-in of functional *FVIII* in a safe harbor locus is another important option for ex vivo gene correction and is useful because of its applicability to monogenic diseases other than hemophilia. Particularly for hemophilia A, such a knock-in strategy can help identify corrective phenotypes by ex vivo therapy in cell types other than LSECs, which do not easily differentiate. Using iPSC models of severe hemophilia, Pang et al. (2016) performed a knock-in of CMV promoter-derived BDD *FVIII* cDNA using transcription activator-like effector nickase targeting an rDNA locus. Although further functional rescue studies are required, this study revealed the potential of rDNA locus as a safe harbor genomic locus. Moreover, Sivalingam et al. (2016) knocked-in a corrective *FVIII* gene in adeno-associated site 1 locus using ZFN fused with an enhanced *Fok*I-Sharkey domain, and reported the secretion of stable FVIII protein in human umbilical cord-lining epithelial cells and bone marrow-derived stromal cells. Some of the above-mentioned studies detected off-target events that could be resolved in the future, and the approaches’ potential efficacy in animal models should be tested extensively. Nevertheless, ex vivo gene correction, which can isolate clones without off-target mutations via genome-wide analysis, provides a promising avenue for clinical studies.

## Conclusion

The application of nucleases technology to precisely induce DSBs at a desired genomic locus enables effective targeted gene editing that could be used to treat genetic diseases. For example, the Sangamo Biosciences research group is preparing a clinical trial on ZFN-mediated in vivo genome-editing in severe-case hemophilia B patients (https://clinicaltrials.gov/ct2/show/NCT02695160), from which we expect to see a significant therapeutic effect. However, the technique’s editing efficiency, frequency of unexpected mutations, and adverse effects remain unknown, and the safety of this approach must be resolved prior to its regular application in human patients. Because phenotypic correction of hemophilia can be achieved with a low corrective efficiency, a strategy for minimizing unexpected mutations should also be explored. Thus, modified Cas9 with highly enhanced target specificity could be very useful in clinical genome-editing (Slaymaker et al. 2016; Kleinstiver et al. 2016). The recently reported cytidine deaminase-fused Cas9 version that edits specific base without DSBs can be considered as another option to correct specific point mutations, potential causes of hemophilia (Komor et al. 2016). Also, the use of mRNA or protein for the nucleases could effectively reduce off-target activities and limit cell-mediated immune responses (Kim et al. 2014; Hendel et al. 2015), although these strategies for in vivo delivery need further improvement. In addition, unbiased genome-wide analysis should be utilized to detect off-target mutations (Bolukbasi et al. 2015), especially methods for identifying low-frequency off-target mutations. Finally, keeping pace with the progression of genome-editing technology, understanding/perception of the society and institutional procedures needs to be improved for the further development of the field. Unless there are any ethical issues involved, this technology is considered to be a promising option that gives opportunities for better life to hemophilia patients as well as other genetic disease patients.
